# A usable model of “decathlon winner” cancer cells in triple-negative breast cancer: survival of resistant cancer cells in quiescence

**DOI:** 10.18632/oncotarget.24322

**Published:** 2018-01-25

**Authors:** Balraj Singh, Vanessa N. Sarli, Laura J. Washburn, Milan R. Raythatha, Anthony Lucci

**Affiliations:** ^1^ Department of Breast Surgical Oncology, The University of Texas MD Anderson Cancer Center, Houston, TX, USA; ^2^ Morgan Welch Inflammatory Breast Cancer Research Program and Clinic, The University of Texas MD Anderson Cancer Center, Houston, TX, USA

**Keywords:** hypoxia, metformin, CB-839, cancer evolution, cancer cell quiescence

## Abstract

We previously described a strategy for selecting highly adaptable rare triple-negative breast cancer (TNBC) cells based on their ability to survive a severe and prolonged metabolic challenge, e.g., a lack of glutamine. We hypothesized that metabolically adaptable (MA) cancer cells selected from the SUM149 cell line in this manner have the capacity to survive a variety of challenges that postulated “decathlon winner” cancer cells must survive to succeed in metastasis. These MA cells were resistant to glutaminase inhibitor CB-839, as predicted from their ability to proliferate without exogenous glutamine. They were also resistant to hypoxia, surviving treatment with hypoxia inducer cobalt chloride. Investigating the nature of intrinsic resistance in SUM149-MA cells, we found that 1–2 mM metformin completely inhibited the emergence of MA colonies in SUM149 cells in glutamine-free medium. These highly resistant MA cells grew into colonies upon removal of metformin, indicating that they survived in quiescence for several weeks under metformin treatment. This approach of selecting resistant cells worked equally well with additional TNBC cell lines, specifically inflammatory breast cancer cell line FC-IBC02 and mouse breast cancer cell line 4T07. In both cases, less than 1% of cells survived metformin treatment and formed colonies in glutamine-free medium. The MA cells selected in this manner were significantly more resistant to the chemotherapeutic drug doxorubicin than the parental cell lines. We conclude that our approach may be useful in developing usable models of cancer cell quiescence and therapy resistance in TNBC.

## INTRODUCTION

Cancer is an evolution-like process involving various mechanisms for generating tumor cell diversity and the selective pressures that operate in the body [1–4]. Therefore, we must get better at developing therapies that inhibit cancer evolution. To meet this challenge, we are pursuing a novel approach for modeling the cancer cells that drive cancer evolution. Growing evidence supports the idea that the metabolic state of a cell is an equal partner in a reciprocal relationship with its regulatory state, encompassing genetic and epigenetic regulation [5, 6]. Accumulating evidence also has shown that specific metabolites play critical roles in determining the epigenetic state of a cell by influencing modifications to DNA and histones, suggesting that the metabolic state of a cell is intimately tied to its regulatory state [6]. The hypothesis underlying our work is that progenitor-like cancer cells have a privileged metabolic state that permits cell survival under starvation conditions that would kill most cancer cells. If a highly abnormal cancer cell can survive a prolonged severe metabolic challenge, then this cell can survive most hurdles, including standard therapies and immune therapies. Given these premises, we are developing an *in vitro* model of the most evolvable and resistant “decathlon winner” cancer cells [3] that can be used to discover effective new therapies for cancers that do not respond to currently offered therapies.

Therapy resistance remains a significant problem in cancer, especially in heterogeneous cancers such as inflammatory breast cancer (IBC) and triple-negative breast cancer (TNBC) [7–9]. These heterogeneous cancers are composed of a large percentage of highly proliferative cancer cells and a very small percentage of non-proliferative cancer cells. Current therapies necessarily target the proliferative cells for disease control but often do not affect the non-proliferative cells, which may be the root of the disease. If the initial therapy offered does not target both the proliferative cells and the non-proliferative “root” cells, there is a high likelihood of therapy resistance, recurrence, and metastasis. In some breast cancer patients, therapy-resistant minimal residual disease (MRD) persists in quiescence for years before advancing to proliferative disease [10, 11]. The major goal of our studies is to model the type of cancer cells that persist as MRD.

Currently, cancer therapies are evaluated mainly to assess their effect on proliferation and survival of cancer cells in short-term assays. This approach is not optimal for discovering therapies that eradicate cancer cells that are relatively quiescent or can enter quiescence for prolonged survival under a therapeutic intervention. There is overwhelming evidence that this is an important feature of therapy-resistant cancer cells. Because the current approach to therapy development is ineffective in eradicating the cancer cells that drive the disease and therapy resistance, this approach ends up advancing the therapies that may accelerate disease recurrence and metastasis simply by eliminating their competition within a heterogeneous disease.

We have previously reported that rare metabolically adaptable (MA) cells present in the SUM49 TN-IBC cell line can survive and grow without exogenous glutamine [12]. The SUM149-MA cells are highly resistant to chemotherapeutic drugs and a variety of other targeted therapeutics [13]. Our previous microarray gene expression analysis supports the hypothesis that resistant MA cells are abnormal progenitor-like cells, which have the capacity to generate a tremendous heterogeneity in progeny cells [13]. This is in addition to the genetic mechanisms that are prevalent in cancer cells for generating cellular heterogeneity. Although generally cancer cell lines are not considered good models of tumor heterogeneity, our results indicate a very high capacity for generating cellular heterogeneity in MA cells. Our studies have shown that this capacity becomes more obvious when resistant cells are selected under a challenge, e.g., in a culture medium without glutamine. Our interpretation is that this capacity to generate cellular heterogeneity is not adequately utilized in an artificial complete culture medium; however, it is essential for survival under a realistic body-like challenge and for cancer evolution.

The most impactful stage for incorporating new therapies in breast cancer is in the adjuvant setting after surgery. Therefore, the modeling of MRD-like disease for discovering anticancer therapies will have a big impact in the clinic. It is widely believed that some resistant cancer cells disseminate and arrive at the future sites of metastases much sooner than they can be detected as clinical metastases [3, 10]. The highly resistant cells among these cells are postulated to have a high capacity to survive in a quiescent state. There are no good models of such cells, particularly from the perspective of identifying new therapies that would be effective against such cells. For this study, we hypothesized that, because of their progenitor cell–like nature, SUM149-MA cells possess a strong capacity to enter quiescence. Here we provide evidence in support of this hypothesis, and propose a general strategy for modeling tumor cell quiescence *in vitro*, which can be used for developing effective therapeutic strategies for overcoming resistance.

## RESULTS

### Resistance to hypoxia in SUM149-MA cells

**Figure 1:**
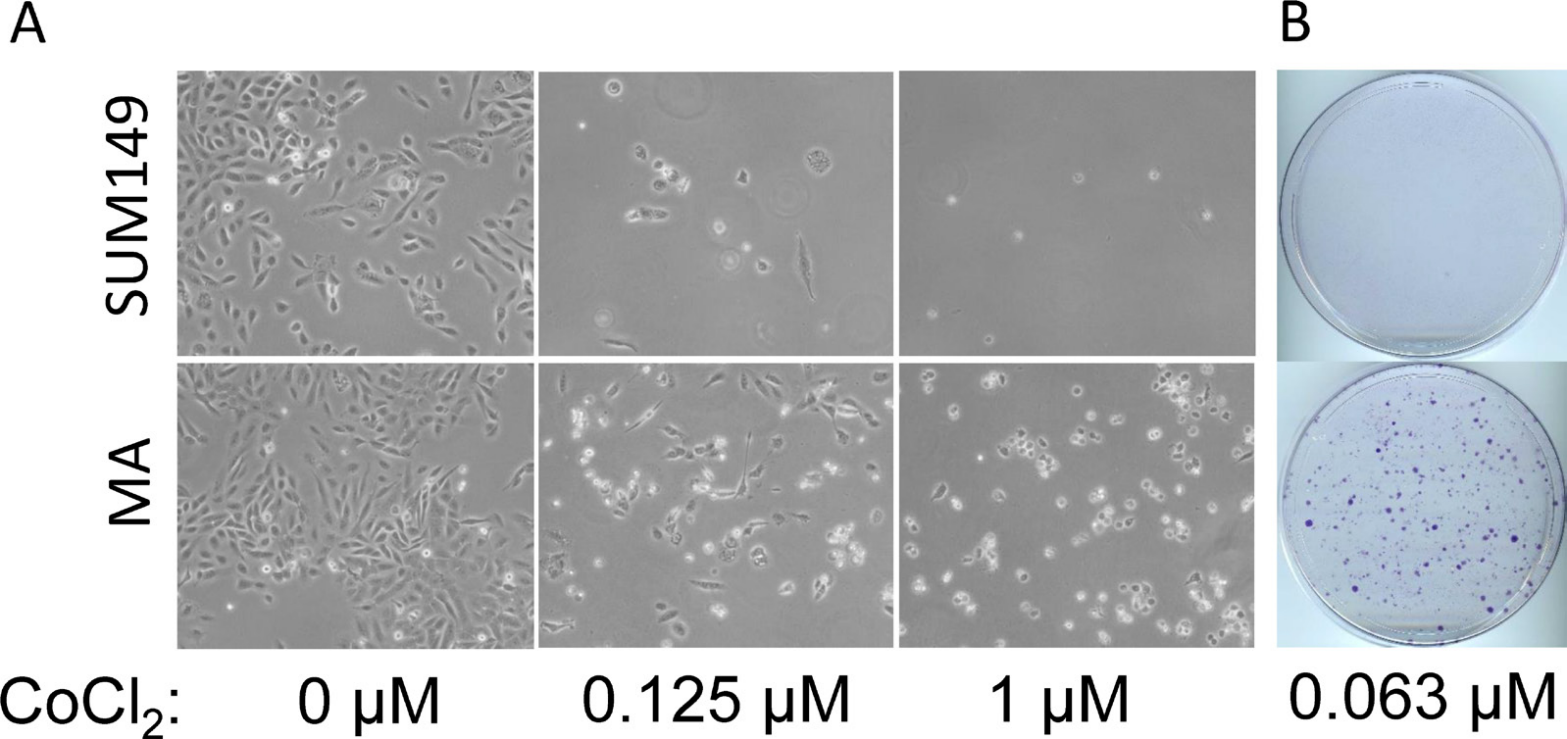
Resistance to hypoxia in metabolically adaptable cells (**A**) SUM149-MA1 cells (MA, bottom) were treated, in parallel with parental luciferase-transfected SUM149 cells (top), with cobalt chloride (CoCl_2_) as a hypoxia mimic. Cells were plated on 10-cm dishes (0.5 million cells per dish) in glutamine-containing medium, and cobalt chloride treatment at the indicated concentrations was initiated the next day and continued for 5 days. The cells were stained with crystal violet, and representative dishes were photographed to document relative cell survival under chemically induced hypoxic conditions. (**B**) SUM149-MA2 cells (MA) were treated, in parallel with parental luciferase-transfected SUM149 cells, with 0.063 µM cobalt chloride for 9 days and then allowed to recover in culture medium without cobalt chloride for 18 days. The resulting colonies were stained with crystal violet.

We previously reported that the rare SUM149 cancer cells that survive a prolonged lack of glutamine in culture medium have progenitor cell–like characteristics [13]. The progeny of these rare cells (0.01% of the SUM149 population) are better at surviving a subsequent prolonged lack of glucose than the parental cell line [13]. One of the well-studied features of resistant cancer cells is their capacity to survive a lack of oxygen. In fact, hypoxia is considered an important element in the evolution of therapeutic resistance. To determine whether SUM149-MA cells are more resistant to hypoxia than the parental SUM149 cell line, we employed cobalt chloride to generate “chemical hypoxia” in cells. This is a widely utilized strategy; cobalt inhibits mitochondrial function and induces hypoxia-related gene expression. We tested the effect of cobalt chloride on parental SUM149 and SUM149-MA cells in a broad concentration range for different lengths of time. Representative cell cultures after 5 days treatment with 0.125 µM or 1 µM cobalt chloride are shown in Figure 1; the MA cells were significantly more resistant to hypoxia than the parental cells. Notably, we chose to analyze these data in a manner that informs about the roots of resistance. We assumed that the majority of cells are sensitive to such insults even in a resistant cell line, and were interested in determining how many cells survive in the resistant cell line when, in a parallel evaluation, 99–100% of cells in the sensitive cell line are killed. This method of evaluating relative resistance is different from traditional cell proliferation/cell toxicity assays that evaluate the effect on the entire cell population. We found that a significantly larger number of MA cells survived treatment with 0.125 µM cobalt chloride for 5 days than SUM149 parental cells, most of which were killed (Figure 1A). Further, treatment that killed all the parental SUM149 cells (1 µM cobalt chloride) failed to kill all SUM149-MA cells (Figure 1A). Cell viability at the end of cobalt chloride treatment in these experiments was confirmed by allowing cells to recover in the medium without cobalt chloride. When we treated parental SUM149 and SUM149-MA cells with 0.063 µM cobalt chloride for 9 days and then allowed them to recover for 18 days in culture medium without cobalt chloride, a large number of the MA cells survived and proliferated to yield colonies, whereas essentially all the parental cells died (Figure 1B). These findings suggest that the MA cells resemble the progenitor-like cancer cells enriched at the core of solid tumors, which can survive deficiencies such as oxygen, glutamine, and glucose.

### Resistance to glutaminase inhibition in SUM149-MA cells

**Figure 2:**
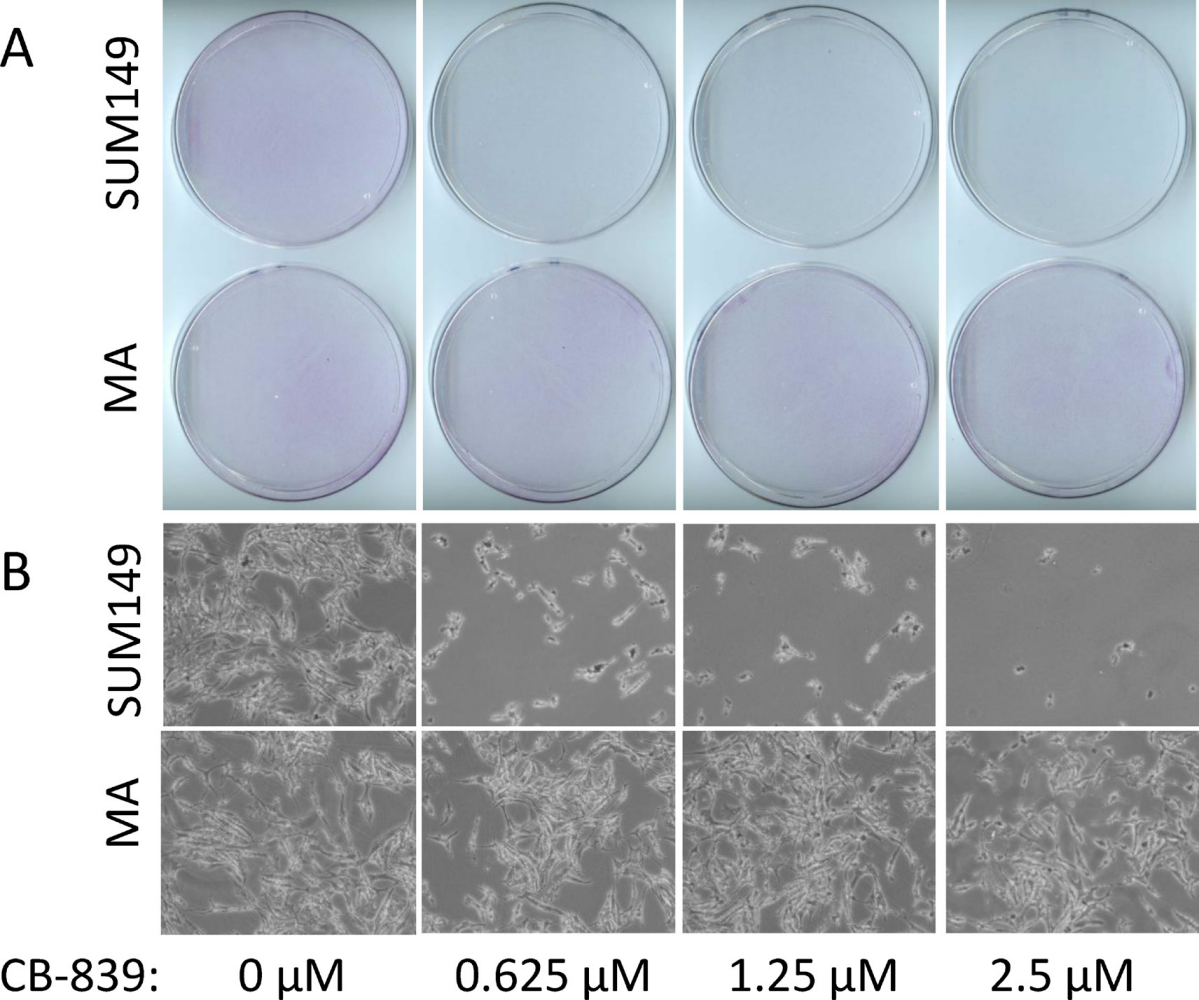
Resistance to glutaminase inhibition in metabolically adaptable cells (**A**) SUM149-MA2 cells (MA, bottom) were treated, in parallel with parental luciferase-transfected SUM149 cells (top), with CB-839, a glutaminase inhibitor. Cells were plated in 10-cm dishes (0.5 million cells per dish) in a glutamine-containing medium, and treatment with the indicated concentrations of CB-839 began the next day and continued for 7 days. The cells were stained with crystal violet, and representative dishes were photographed to document relative cell growth under CB-839 treatment. (**B**) Representative fields in stained dishes shown in panel A were photographed under a microscope.

Glutamine plays an important role in cancer cells besides its role as a building block of proteins [14, 15]. Therefore, strategies are being developed to inhibit glutamine metabolism in cancer cells. One such strategy is inhibition of glutaminase, which catalyzes conversion of glutamine to glutamic acid. The glutaminase inhibitor CB-839 is currently in clinical trials (reviewed in reference [16]). Since SUM149-MA cells survive and grow in glutamine-free medium, and since their expression of glutaminase is lower than in the parental cells [12], we hypothesized that glutaminase may not be important for their survival. To test this, we treated both MA and the SUM149 parental cells with different concentrations of CB-839. Although MA cells were selected and expanded in glutamine-free medium, glutamine was present during the evaluation of CB-839. We found that, as expected from the previous reports on the role of glutamine in TNBC [17], CB-839 severely inhibited the growth of parental SUM149 cells. There was a near 100% inhibition of SUM149 cell growth after treatment for 7 days with 0.625 µM to 2.5 µM CB-839 (Figure 2). In contrast, the same concentrations of CB-839 did not cause any detectable growth inhibition in MA cells; the cells continued to grow similarly to vehicle-treated cells and reached sub-confluency in 7 days (Figure 2). The CB-839–treated parental SUM149 cells exhibited no signs of cell growth, while the growth and morphology of treated SUM149-MA cells appeared unaffected (Figure 2A and 2B).

Because these rare MA cells survive and form colonies in glutamine-free medium, they would be expected to be glutamine-independent and resistant to glutaminase inhibition. However, it takes 4–6 weeks for the emergence and growth of MA cell colonies in a glutamine-free medium to a stainable stage [12, 13]. Parental SUM149 cells were treated with CB-839 for only a week; therefore, we are unable to conclude whether the resistance to CB-839 observed in the SUM149-MA cells pre-exists in rare parental cells and/or develops/matures under glutamine deficiency.

In considering the basis of CB-839 resistance in SUM149-MA cells, we conclude that a severe metabolic challenge, e.g., a prolonged lack of glutamine, would force the selection of a plastic metabolic state which is intertwined with a plastic regulatory state [12, 13, 18]. This would be the basis of selection of progenitor-like cancer cells in our system. Once such cells are selected, they can utilize a variety of mechanisms for cell survival and growth depending upon the surrounding conditions. If this is the case, the metabolic state partners with the genetic and epigenetic landscape to determine cell fate. To survive and eventually grow in a glutamine-free medium, as in this example, a capacity to reduce glutaminase activity and to increase glutamine synthase activity may be important. Indeed, the SUM149-MA cells express a lower level of glutaminase and a higher level of glutamine synthase than the parental cell line [12] (gene expression data in reference [13]). These specific features of metabolic state (plus other ways of generating sufficient glutamine, e.g., metabolite-mediated allosteric enzyme regulation), along with a high overall adaptability/plasticity, would enable SUM149-MA cells to be resistant to CB-839.

Recent studies investigating the cancer cells in less perfused regions of heterogeneous cancers support our interpretation regarding the nature of resistance in MA cells [19]. These studies show that if cancer cells do not receive sufficient glutamine, e.g., at the core of growing tumors, they can generate only a low level of alpha-ketoglutarate, an important regulator of epigenetic state. The lack of glutamine at the core of tumors selects for an undifferentiated, more progenitor-like cell state [19]. Thus our approach offers the opportunity of modeling the type of intrinsic resistance that evolves in cancer in the body.

### Metformin halts the emergence of MA cell colonies

In a search for therapeutic agents that may affect resistant MA cells, we chose to evaluate metformin for several reasons, including 1) reports of metformin affecting “cancer stem cells” [20]; 2) its possible perturbation of the regulation of AMP kinase, which plays a key role in the coordination of cellular response to a metabolic challenge; and 3) its inhibition of slow-growing resistant cancer cells by inhibiting mitochondrial electron transport chain complex I [21]. While highly proliferative cancer cells rely heavily on aerobic glycolysis, more resistant—often quiescent—cells depend more on oxidative phosphorylation for ATP production [22]. Besides this scientific rationale for using metformin, there is an attractive possibility of being able to re-purpose this diabetes drug for cancer therapy.

First, to determine whether metformin interferes with the survival and growth of MA cells in a glutamine-free medium, we shifted SUM149 cells to a glutamine-free medium with or without metformin for several weeks. As previously reported, more than 99% cells died quickly (within 2 days) without glutamine [12]. However, it took approximately 5 weeks for the surviving cells to yield stainable colonies; periodic examination of cell cultures under a microscope during this period revealed that surviving cells encountered different fates. Some cells did not divide and died; other cells underwent different degrees of cell division as indicated by clusters of few cells or tiny colonies, but many of these cells failed to yield healthy colonies and most such tiny colonies disappeared through cell death. It is possible that some cells survived for 5 weeks but failed to grow; it could be argued that some such cells also may persist in the body. The cells that succeeded in growing into colonies could presumably have undergone epigenetic and genetic changes to survive a severe and prolonged metabolic challenge. Although we have reported on the gene expression changes and on the gene amplifications and gene deletions in MA cells [13, 18], it is not possible to know which of these pre-existed and which occurred during selection by prolonged lack of glutamine. *In vitro* cell culture experiments are typically suitable for investigating proliferating cells, but not the cells that may survive in quiescence.

**Figure 3:**
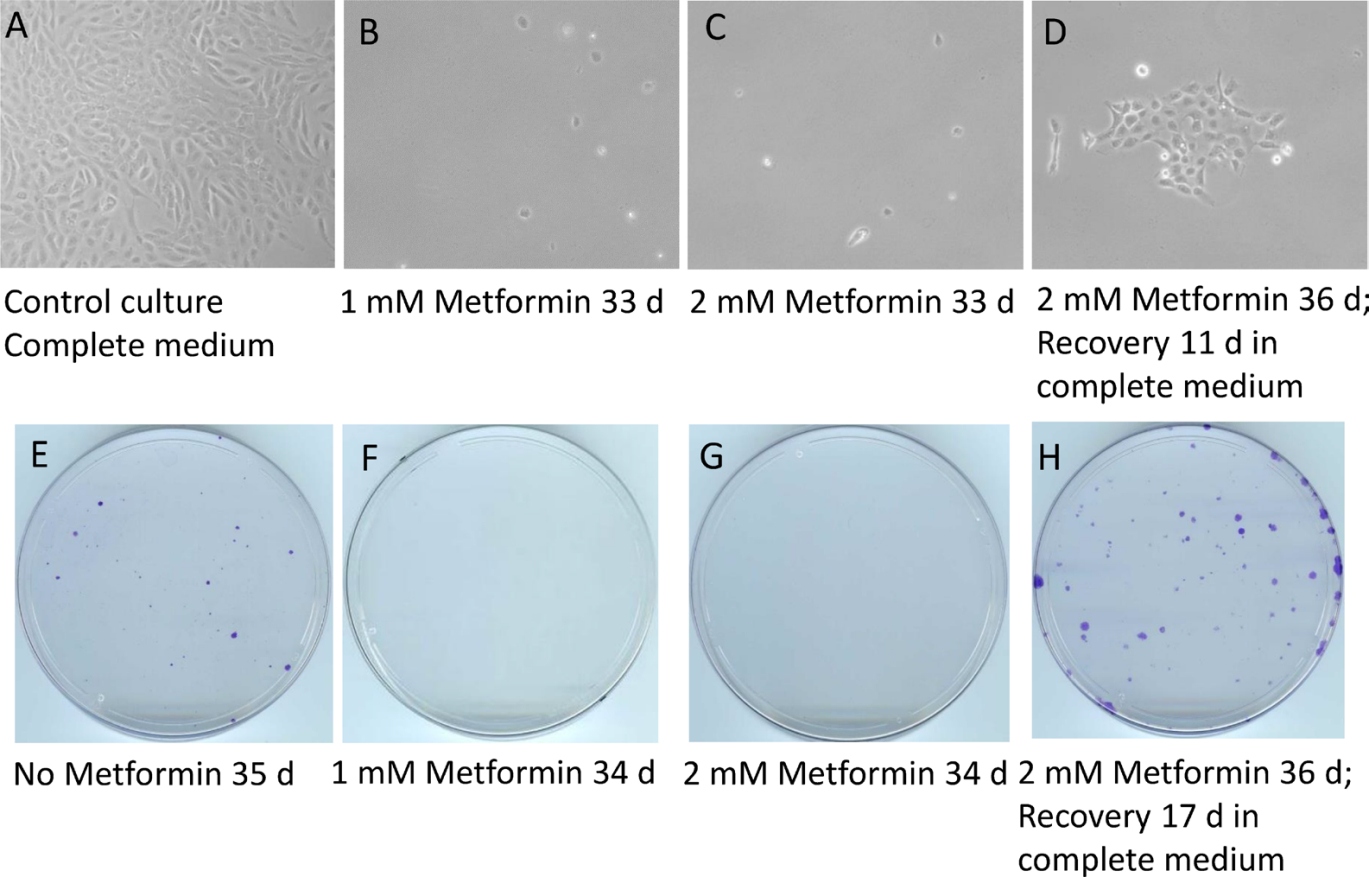
Metformin inhibits the emergence of cell colonies in glutamine-free medium but does not eradicate quiescent cancer cells Luciferase-transfected SUM149 cells were plated in 10 cm dishes (0.5 million cells per dish) in glutamine-free medium. The next day, metformin was added as indicated (1 mM or 2 mM) and this treatment continued for 33–36 days. The cells in the dishes were stained with crystal violet. The top panel (**A–D**) shows representative fields from these dishes after various treatments, photographed under a microscope. The bottom panel (**E–H**) depicts photographs of entire representative dishes. (A, E) Control cells grown in complete medium for 7 days (A) or in glutamine-free medium without metformin for 35 days (E). (B, F) Cells treated with 1 mM metformin in glutamine-free medium for 33 or 34 days. (C, G) Cells treated with 2 mM metformin in glutamine-free medium for 33 or 34 days. (D, H) Cells treated with 2 mM metformin in glutamine-free medium for 36 days and then allowed to recover in complete medium without metformin for another 11 days (D) or 17 days (H). Although not clearly evident from the image, the dish treated with 1 mM metformin (F) had one small colony; there were no colonies in the dish treated with 2 mM metformin (G). Comparison of C and G with D and H demonstrates that metformin treatment arrested colony growth but did not eradicate the quiescent cancer cells.

Of SUM149 cells treated with 2 mM metformin without glutamine, like those treated with vehicle, more than 99% died within 2 days. As we periodically monitored the rare surviving cells under a microscope, we noticed that metformin had a strong growth-inhibitory effect. It prevented cells from dividing; all cells stayed as single cells, most of which subsequently died. When the dishes were stained after about 35 days of culture (Figure 3A–3D), no colonies were detected in the cultures treated with metformin, not even tiny ones or cell clusters of any size. The cultures treated with vehicle (controls) each had approximately 20 colonies easily visible and approximately 20 tiny colonies (Figure 3E). Treatment with a lower dose (1 mM) of metformin also caused a near-complete inhibition of colony growth (Figure 3F–3H).

Notably, 1–2 mM metformin had a growth-inhibitory effect in SUM149 cells growing in glutamine-free medium, but not in those growing in complete medium (Supplementary Figure 1). Although metformin slightly reduced the growth of cells (compare panels in Supplementary Figure 1), they continued to grow for multiple passages. Presumably, TNBC cells do not have sufficient redundancy of pathways to simultaneously survive both metformin and a metabolic challenge (e.g., lack of glutamine). The cells may have more choices of metabolic pathways for their survival and growth under metformin treatment in the complete medium. The most important difference between glutamine-free medium and complete medium is that 99.99% of cells in glutamine-free medium were eliminated by cell death. Therefore, what these results may show is that the effect of metformin varies in different subpopulations and in different culture conditions. Our data suggest that the 0.01% of cells that survive and grow in glutamine-free medium are progenitor-like and resemble the type of cells that drive resistance in the body [13]. Therefore, metformin’s observed inhibitory effect on the emergence of MA cell colonies is significant.

Next, we asked whether metformin would inhibit growth of SUM149-MA cells after they have been selected in glutamine-free medium and passaged several times in this medium. We found that, once established as cell cultures in this manner, SUM149-MA cells were no longer sensitive to the growth-inhibitory effect of 1–2 mM metformin irrespective of the glutamine content of the culture medium (Supplementary Figure 1). Although metformin slightly reduced the growth of cells, they continued to grow for multiple passages (Supplementary Figure 2). One way to explain metformin’s inhibitory effect on the emergence of initial colonies in glutamine-free medium versus its lack of significant inhibitory effect in established SUM149-MA cultures in the same medium is the difference in their epigenetic state. Our data suggest that metformin is effective in inhibiting cell growth at the early stage (when rare cells are possibly reprograming their epigenetic state to survive and to grow under a selection pressure) leading to colony formation, but not after the establishment of a stable cell line.

### Metformin does not kill progenitor-like cancer cells

Although metformin treatment was very effective in inhibiting the emergence of colonies in glutamine-free medium, we could not discern whether it had eliminated all cancer cells. It is possible that metformin simply stopped the cells from dividing. Morphological analysis of the rare single cells on the dish did not indicate whether a cell was dead or alive, let alone predict whether it could form a colony. To determine whether SUM149 cells treated with metformin in glutamine-free medium for 36 days retained colony-forming capacity, we allowed cells to recover and grow in complete medium lacking metformin. We noticed that a good number of these cells yielded colonies; a dish stained after 17 days of recovery had approximately 50 visible colonies of different sizes (Figure 3H). The number of colonies after recovery was approximately the same as observed after 5 weeks of selection in glutamine-free medium without metformin (compare panels E and H in Figure 3), indicating that all progenitor-like cells may have survived metformin treatment as single cells in quiescence for 36 days and that they maintained their ability to grow into colonies.

In a similar experiment, we allowed recovery of metformin-treated cells in glutamine-free medium or in complete medium. We observed growth of colonies in both media. However, colonies grew faster and to a larger size in complete medium than in glutamine-free medium. Typically, we observed a larger number of colonies growing in complete medium than in glutamine-free medium (data not shown). It is possible that these colonies represent cells that can survive without glutamine but still need glutamine for growth. Alternatively, some colonies that grow in glutamine-free medium could be so small that they are not visible on stained dishes. It is conceivable that there are two different subpopulations of SUM149 cells that survive in glutamine-free medium: one that can grow without glutamine and the other that cannot. Metformin does not appear to diminish the survival of either subpopulation; it merely inhibits their growth. Our results imply that metformin has a potential to inhibit TNBC progression in patients with MRD, who are at a high risk of recurrence and metastasis. However, metformin’s efficacy may be limited after TNBC has advanced to clinical metastasis.

### Generalizability of approach for modeling therapeutic resistance

**Figure 4:**
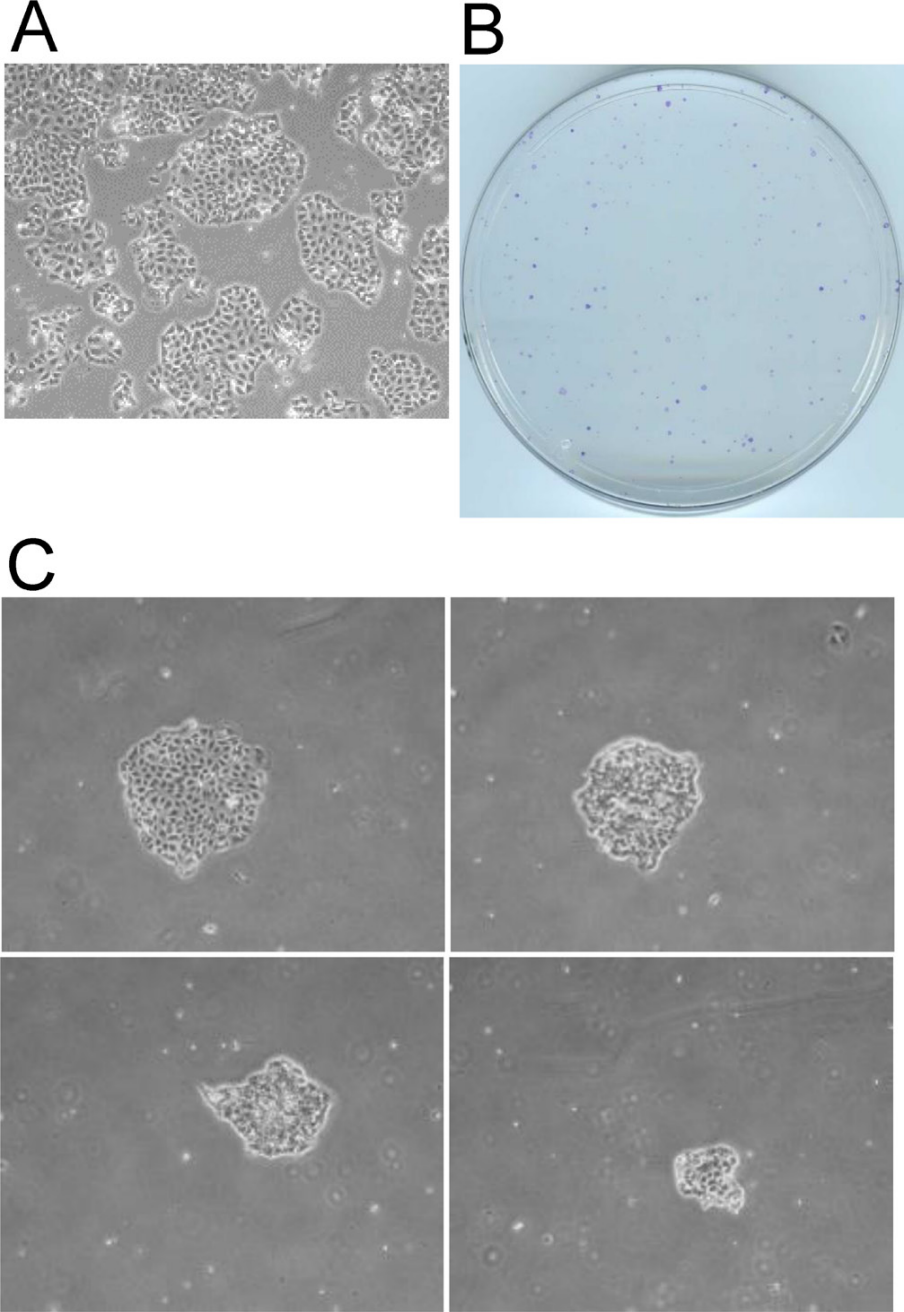
Selection of metabolically adaptable cells from the FC-IBC02 cell line FC-IBC02-MA cells were selected in glutamine-free DMEM. (**A**) FC-IBC02 cells were plated on culture dishes in complete medium and allowed to grow for 6 days. A representative field from one dish is shown. (**B**, **C**) Cells were plated in 10 cm culture dishes (1 million cells per dish) in glutamine-free medium and maintained for 43 days. (B) The dishes were stained with crystal violet and photographed. The growth of rare surviving cells (approximately 0.01% of the total FC-IBC02 population). (C) Four MA cell colonies of different sizes photographed under a microscope.

Although we used the very resistant TN-IBC SUM149 cells to deepen our understanding of the roots of resistance, we tested our observations in other cell lines. Repeating these experiments in a recently developed TN-IBC cell line FC-IBC02 [23], and in mouse breast cancer cell line 4T07 (for its suitability for use in a syngeneic mouse model) yielded results similar to those obtained with SUM149 cells. Lack of glutamine quickly killed the majority of FC-IBC02 cells, whereas rare cells (0.01% of the total population) survived and grew, yielding a stable line of MA cells that formed colonies (Figure 4). We previously described the selection and growth of 4T07-MA cells in glutamine-free medium; MA cells were referred to as glutamine-independent (Gln-ind) in that study [12].

**Figure 5:**
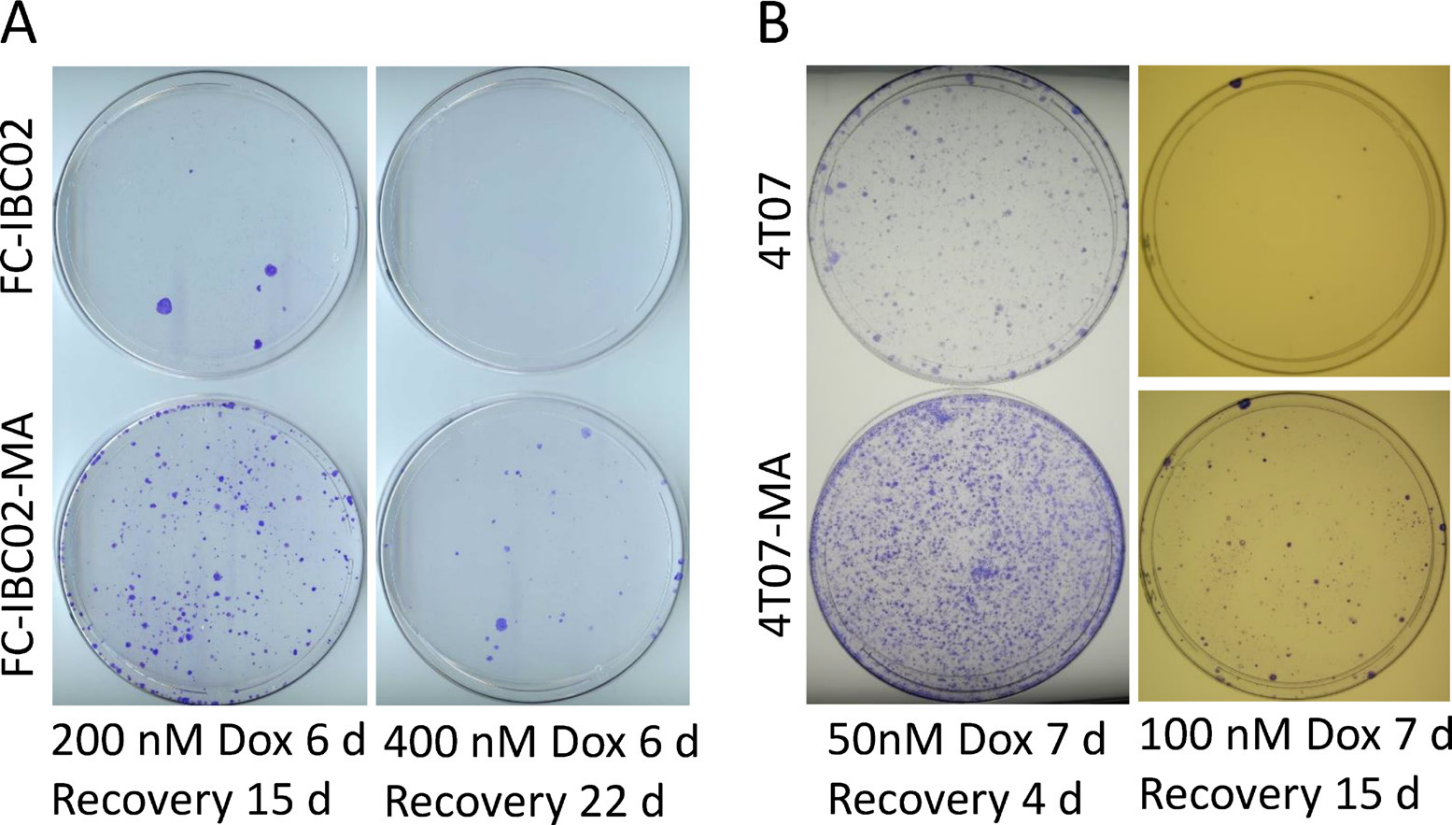
FC-IBC02-MA and 4T07-MA cells are relatively more resistant to doxorubicin than their parental cells FC-IBC02-MA and parental FC-IBC02 cells (**A**) and 4T07-MA and parental 4T07 cells (**B**) cells were treated with the indicated concentrations of doxorubicin (Dox) for 6–7 days in glutamine-containing medium. The doxorubicin was then washed off and the surviving cells were allowed to recover and grow into colonies for the indicated periods. The dishes were stained with crystal violet.

From the perspective of developing an *in vitro* model of resistance, the MA cells would be less interesting if they were not significantly resistant to a chemotherapy drug such as doxorubicin. It is possible to select variant cells that can survive glutamine deficiency (because their metabolism is altered to generate sufficient glutamine) without being highly resistant; an example would be MCF7 cells that can overcome glutamine deficiency since they produce a high amount of glutamine synthase [24]. Therefore, we evaluated the efficacy of doxorubicin in eradicating resistant FC-IBC02-MA and 4T07-MA cells in parallel with their parental cells. We treated cells with different concentrations of doxorubicin for 6–7 days (a treatment sufficient to kill 99% of parental cells), then allowed the surviving resistant cells to grow into colonies for 15 to 22 days to determine relative resistance. The results indicate that both types of MA cells were significantly more resistant to doxorubicin than the parental cells, forming at least 10 times more colonies than their parental cells (Figure 5).

To address whether metformin also inhibits the emergence of FC-IBC02 colonies in glutamine-free medium as observed with the SUM149 cells, we performed similar experiments. The results show that 2 mM metformin inhibited the formation of FC-IBC02 colonies in glutamine-free medium. Furthermore, as in SUM149 cells, metformin treatment arrested rare FC-IBC02 cells in quiescence, which grew into colonies in either glutamine-free medium or complete medium upon removal of metformin (Supplementary Figure 3). Two different TN-IBC cell lines responding similarly to glutamine deficiency and to metformin treatment offers hope that our approach is suitable for establishing models of MA cells from other heterogeneous cancers, including metastatic cancers.

Although the approach of selecting resistant cells based on their metabolic adaptability may have broad applicability, the selected cells will likely differ in the molecular networks that govern their adaptability. For these cells to be realistic models of resistant disease, we prefer the ones that are capable of generating a high degree of cellular heterogeneity, such as the SUM149-MA cells. If we are able to identify therapeutic agents that appear promising against SUM149-MA cells, it would be useful to test them on additional MA cell lines, e.g., FC-IBC02-MA and 4T07-MA, to gain confidence about their efficacy as the agents move toward testing in patients. By deliberately modeling cells that generate a high degree of genetic and epigenetic heterogeneity, and by testing therapeutic agents in long-term assays that reveal whether the roots of resistance are affected, we will have a better chance of predicting the fate of new therapies in patients.

## DISCUSSION

### Proposed *in vitro* model of quiescent cancer cells

The cancer cells with a high ability to survive in quiescence are often responsible for resistance to therapies. Therefore, there is a pressing need for modeling such cells for evaluating therapies that would eradicate such cells. Such evaluations will complement widely used approaches to assess therapeutic efficacy, such as growth inhibition and/or death of proliferating cancer cells. Our results presented here suggest that metformin treatment in glutamine-free medium could be used to arrest highly adaptable progenitor-like cancer cells in quiescence for several weeks. Since these cells maintain their colony-forming capacity, they could be used for evaluating potential therapeutic agents that affect cells in quiescence. The general strategy would be to treat quiescent cancer cells with single agents or combination therapies for several weeks, and then evaluate the treated cells for colony-forming capacity and chemotherapeutic resistance. To utilize this approach, we must know the time period during which metformin-treated cells stay healthy. We have noticed that very long treatment (2 months or more) with metformin affects cells’ capacity to form colonies. We suggest that it may be possible to lengthen the period of quiescence by making changes to the culture medium. It may also be possible to use other agents instead of metformin to arrest resistant cells in quiescence. At minimum, our results suggest that even when cancer cells are maintained in artificially rich culture medium, they may maintain a small subpopulation of cells that can survive in quiescence rather than die when nutrients become scarce.

How does the quiescence modeled in our *in vitro* system relate to cancer cell quiescence in TNBC patients? At the outset, the TN-IBC cell lines used here may be viewed as a model of aggressively proliferating cancer cells not only because *in vitro* culture is inherently optimized for cell proliferation, but also because the cell lines were established from very resistant cancers. While that may be true, we hypothesize that an ability to survive in quiescence when faced with extreme adversity (including therapies) may be a defining characteristic of progenitor-like decathlon winner cancer cells. Although decathlon winner cancer cells may not be “visible” in proliferating cells in rich culture medium, our results suggest that they are enriched in glutamine-free medium wherein 99.99% cells die. Even when these cells reprogram to proliferate under the condition they find themselves in, they maintain a small subpopulation of progenitor-like cells that has a high ability to enter quiescence when faced with a new challenge.

### Model of intrinsic resistance in TNBC

**Figure 6:**
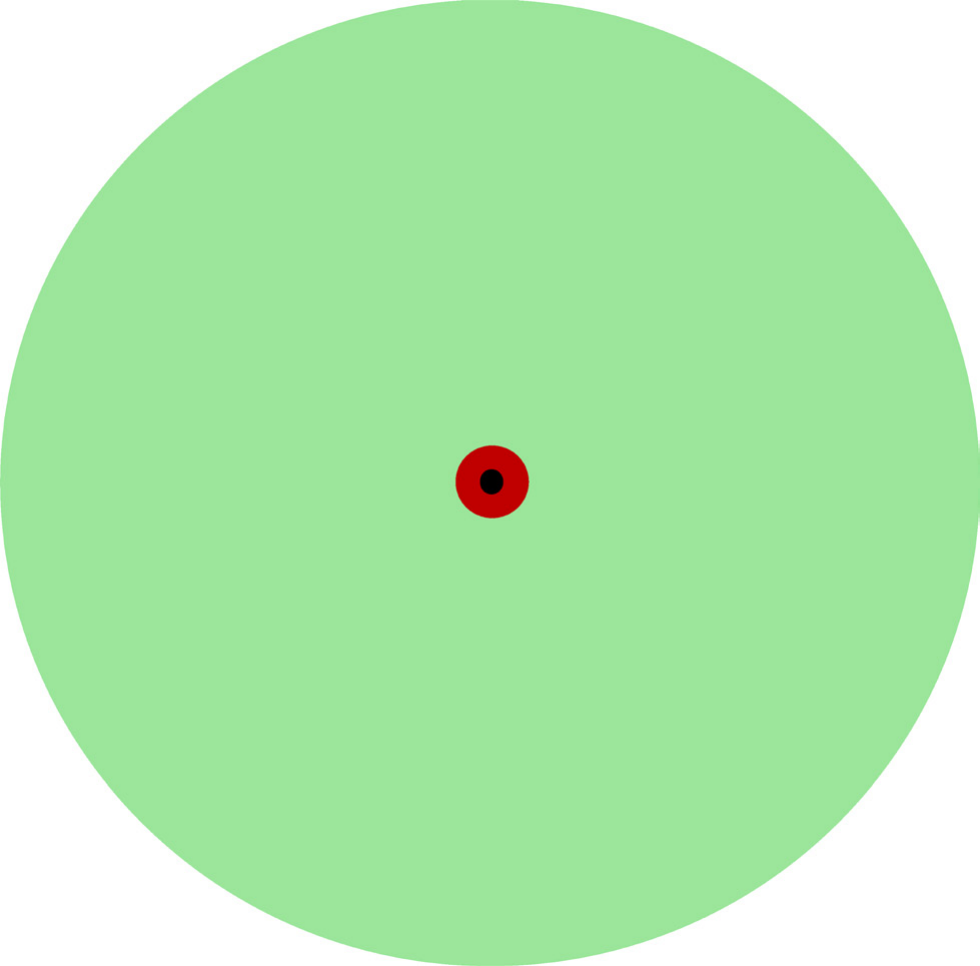
A hierarchical model of therapeutic resistance in TNBC This model is helpful in explaining our results obtained with MA cells. Most cancer cells (green) are sensitive to current therapies, but about 1% of cells (red) develop resistance to monotherapies, and about 0.01% of cells (black) have high resistance to multiple therapies. There is a dynamic equilibrium between these subpopulations. Our approach is focused on developing a usable *in vitro* model of highly resistant cancer cells. Not drawn to scale.

In our working model, cancer cells in a heterogeneous tumor fall into one of three categories: highly resistant cells comprising about 0.01% of cancer cells; resistant cells comprising about 1% of cancer cells; and sensitive cells comprising about 99% of cancer cells (Figure 6). This model is more appropriate for explaining our results with the MA cells than the conventional models involving two subpopulations of cancer cells, e.g., cancer stem cells versus non-cancer stem cells or resistant cancer cells overexpressing a driver versus the rest of cancer cells. We are modeling highly resistant cancer cells into usable *in vitro* systems for discovering therapies that will overcome intrinsic resistance and improve outcomes. Our *in vitro* approach has flexibility for modeling body-like selection pressures in the context of high intracellular heterogeneity and evaluating therapeutic agents over long periods. These features can be utilized for improving the predictability of tumor responses when new therapeutic agents move to clinical trials in TNBC.

### Relevance of the *in vitro* model in the clinic

The most important issue for our *in vitro* model is its relevance to the resistant cancer cells in TNBC patients. To qualify as a decathlon winner cancer cell, a cell must possess a high intrinsic resistance. Extrinsic factors (e.g., immune system, metabolic challenges, therapeutic drugs) in the body control the fate of cancer cells: while a large majority of cancer cells can be eliminated, decathlon winner cells persist (often in quiescence) and eventually proliferate [3]. Even though it is not feasible to model all the extrinsic factors that operate in the body, we can model a high intrinsic resistance as described here. To render the resistant cells more body-like and heterogeneous, we applied a severe metabolic challenge that is known to operate in the body [19]. The *in vitro* model of MA cells normally features proliferating cells; however, we show here that highly resistant cells have a strong capacity for surviving in quiescence. Both of these models (MA cells in quiescence and MA cells after they adapt to proliferate) can be utilized for improving drug discovery efforts for TNBC, wherein disseminated tumor cells exist predominantly in quiescence prior to recurrence/metastasis and in proliferative phase as disease progresses. It could be argued that since intrinsic resistance does not depend on extrinsic factors by its very definition, a lack of extrinsic factors may not be a cause of concern if the major goal is to model intrinsic resistance. On the contrary, it gives a freedom to model intrinsic resistance without the extrinsic factors that are likely to vary anyway depending upon the organ. We can utilize the unique strengths of an *in vitro* model, e.g., an ability to test a variety of therapeutic compounds in long-term assays, for optimally modeling intrinsic resistance for drug discovery.

## MATERIALS AND METHODS

### Cell lines and culture

The SUM149 TN-IBC cell line, originally obtained from Stephen Ethier (Barbara Ann Karmanos Cancer Institute, Detroit, MI, USA), was grown in Ham F-12 medium supplemented with 5% fetal bovine serum (FBS), 5 µg/mL insulin, 1 µg/mL hydrocortisone, 100 U/mL penicillin, and 100 µg/mL streptomycin in a humidified 5% CO_2_ atmosphere. We previously described SUM149-Luc, the luciferase-transfected SUM149 cell line [25]. The FC-IBC02 TN-IBC cell line, originally developed by Massimo Cristofanilli [23], was obtained from the cell line collection of the Morgan Welch Inflammatory Breast Cancer Research Program and Clinic. It was grown as an adherent culture in Dulbecco modified Eagle medium (DMEM) supplemented with 10% FBS. The 4T07 mouse breast cancer cell line, originally isolated in the laboratory of Fred Miller [26], was obtained from Ralph Arlinghaus (MD Anderson Cancer Center) and grown in DMEM/F-12 medium supplemented with 10% FBS.

### Selection and culture of MA cancer cells

We selected MA cells by plating 1 million SUM149-Luc cells in a glutamine-deficient medium containing dialyzed FBS (to reduce the level of glutamine in the medium to near 0). We recently described selection of these rare cells (approximately 0.01% of the total SUM149 population) and characterization of a cell culture established from these colonies [12, 13]. We have two MA cell lines: MA1, developed from initial colonies selected from 0.5 million SUM149-Luc cells, and MA2, developed from initial colonies selected from one million SUM149-Luc cells [12, 13]. MA cells can be passaged indefinitely in glutamine-deficient medium. However, to minimize the loss of cellular characteristics in cell culture, we used MA cells that were in a glutamine-free medium for less than 10 passages. Similarly, for investigating the behavior of MA cells in a glutamine-containing medium, we used cells cultured for less than 10 passages.

Selection of the rare MA cell variants from the FC-IBC02 cell line was similar to the selection of the SUM149-MA variants, except that glutamine-free DMEM supplemented with 10% dialyzed FBS was used for selection and initial growth of colonies as stable cultures for several passages. We similarly selected and established the rare MA cell variants in culture from the 4T07 cell line in glutamine-free DMEM/F-12 medium supplemented with 10% dialyzed FBS [12].

### Reagents and drugs

The drugs used in this study were purchased from commercial sources: CB-839 from Selleck Chemicals (Houston, TX) and cobalt chloride, metformin, and doxorubicin from Sigma-Aldrich (St. Louis, MO). We dissolved CB-839, and doxorubicin, in dimethyl sulfoxide (DMSO) and dissolved metformin in water. We added an equal volume of the solvent to all dishes, including the control dishes not treated with a drug. DMSO volume was 0.04% of the volume of the culture medium.

### Assay of relative resistance to chemotherapeutic drug doxorubicin

We typically plated 0.5 million MA cells or corresponding parental cells in each 10-cm dish with culture medium containing glutamine. After 24 hours, doxorubicin (50–400 nM) dissolved in DMSO was added. Drug treatment continued for 6–7 days while response to the drug was monitored under a microscope; this treatment typically killed the majority of cells, leaving behind the most resistant cancer cells. The drug-containing medium was removed, and the cells were washed twice with phosphate-buffered saline solution and incubated in the glutamine-containing medium without drug for several days to weeks until colonies visible to the naked eye appeared. We stained the colonies with crystal violet and photographed or scanned the cultures.

## CONCLUSIONS

Simple and reliable models are urgently needed for testing potential therapeutic drugs that might overcome intrinsic resistance to therapies in heterogeneous cancers such as TNBC. A variety of potential therapeutic agents, e.g., those affecting epigenetic programing in rare but highly resistant cancer cells, need to be tested for long periods to determine their efficacy. Our usable model is designed to evaluate monotherapies and combination therapies for long periods, to reveal the nature of response versus resistance. The data presented in this paper suggest the existence of highly resistant TNBC cells that can enter quiescence to survive a metabolic challenge (and possibly other challenges because of their progenitor-like plastic state). Thus our approach can be translated into usable models of both quiescent and proliferating cancer cells that are responsible for therapy resistance. Potential therapeutic agents that will be effective in eradicating or disabling the most resistant MA cancer cells will improve patient outcomes by affecting the roots of the disease.

## SUPPLEMENTARY MATERIALS FIGURES



## Abbreviations

DMEM: Dulbecco modified Eagle medium
DMSO: dimethyl sulfoxide
FBS: fetal bovine serum
IBC: inflammatory breast cancer
MA: metabolically adaptable
MRD: minimal residual disease
TNBC: triple-negative breast cancer
TN-IBC: triple-negative inflammatory breast cancer
