# Supplementary material for: A usable model of “decathlon winner” cancer cells in triple-negative breast cancer: survival of resistant cancer cells in quiescence
Source: Oncotarget. 2018 Jan 25;9(13):11071–82. doi: 10.18632/oncotarget.24322 (PMC5834289; doi:10.18632/oncotarget.24322)
Supplement: Supplementary file 1 [file oncotarget-09-11071-s001.pdf]

A usable model of "decathlon winner" cancer cells in triple-negative breast cancer: survival of resistant cancer cells in quiescence

SUPPLEMENTARY MATERIALS

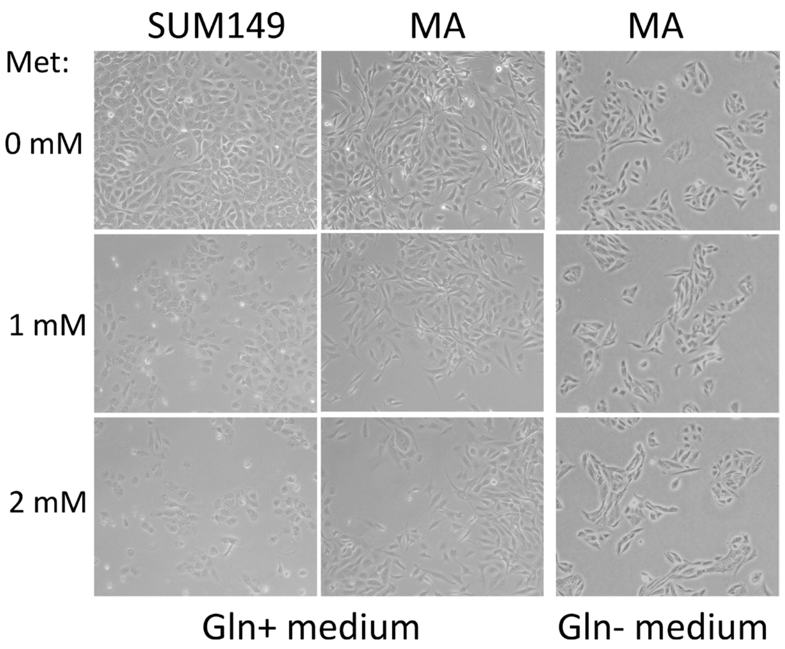

Supplementary Figure 1: Metformin has relatively little effect on SUM149 cells in medium containing glutamine or in SUM149-MA cells in medium with or without glutamine (related to Figure 3). SUM149-Luc cells (left) and SUM149-MA cells (middle) were plated and treated in parallel with indicated doses of metformin (Met) for 7 days in glutamine-containing (Gln+) medium. SUM149-MA cells also were plated and grown with indicated doses of metformin for 7 days in glutamine-free (Gln–) medium (right). Representative fields of cells were photographed under a microscope. The modest growth inhibition under each of these conditions contrasts sharply with the severe inhibition of colony growth when metformin was added at the time of initial selection of MA cells (Figure 3). Also see Supplementary Figure 2.

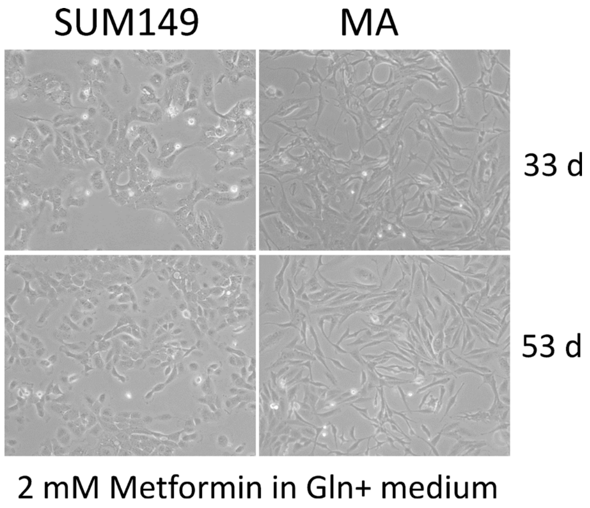

Supplementary Figure 2: SUM149 and SUM149-MA cells after long treatment with 2 mM metformin (related to Figure 3). SUM149-Luc cells (left) and SUM149-MA cells (right) were plated and grown in parallel with 2 mM metformin for 33 days (top) or for 53 days (bottom) in glutamine-containing (Gln+) medium. Representative fields of cells were photographed under a microscope. Number of passages before 33 days: SUM149-Luc, 2; SUM149-MA, 3. Number of passages before 53 days: SUM149-Luc, 5; SUM149-MA, 6. The relatively normal growth rates and normal cell morphologies after these long treatments with 2 mM metformin indicate that both these cell lines can fairly quickly adapt to metformin treatment.

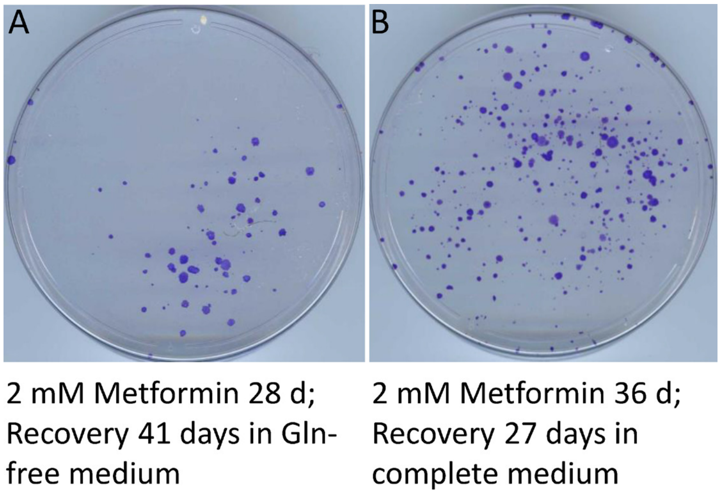

Supplementary Figure 3: Metformin treatment maintains highly resistant FC-IBC02 cells in quiescence in glutamine-free medium (related to Figure 4). FC-IBC02 cells were plated in 10 cm culture dishes (1 million cells per dish) and treated with 2 mM metformin in glutamine (Gln)-free medium for 28 days (**A**) or 36 days (**B**). Microscopic evaluation revealed a total lack of colonies of any size after either time period. We then washed off the metformin and allowed the cells to recover in a glutamine-free medium for 41 days (A) or in complete medium for 27 days (B). We observed a significant number of colonies in both conditions, indicating that metformin merely caused a reversible growth arrest in metabolically adaptable cancer cells.
